# A Rare Pseudotumoural Bladder Presentation Revealing Crohn’s Disease in the Absence of an Enterovesical Fistula: A Case Report

**DOI:** 10.7759/cureus.107787

**Published:** 2026-04-27

**Authors:** Anass El Alaoui, Anouar El Moudane, Achraf Benamou, Mohamed Moukadem, Ali Barki

**Affiliations:** 1 Urology, Mohammed VI University Medical Center, Faculty of Medicine and Pharmacy, Mohammed the First University, Oujda, MAR

**Keywords:** crohn’s disease (cd), enterovesical fistula, haematuria, inflammatory bladder tumor, pseudotumoral bladder

## Abstract

Urological involvement in Crohn's disease is rare and most often associated with enterovesical fistulas. However, reactive thickening of the bladder wall without true fistulous communication is rarely described and may suggest a malignant bladder tumour. We report the case of a 63-year-old man presenting with lower urinary tract symptoms and gross haematuria. The results of imaging and cystoscopy initially suggested a bladder tumour. Further examinations revealed fistulous Crohn's disease with an inflammatory collection above the bladder causing marked reactive thickening of the bladder dome, with no sign of enterovesical fistula. This case highlights an unusual presentation of Crohn's disease and underlines the importance of considering extrinsic inflammatory causes in the differential diagnosis of bladder pseudotumours.

## Introduction

Crohn's disease is a chronic inflammatory bowel disease that can affect any segment of the digestive tract and is often complicated by the formation of fistulas. Urological manifestations are rare and usually result from enterovesical fistulas, occurring in approximately 2-5% of cases, mainly in men [1].

Contiguous involvement of the bladder without a direct fistula is exceptional and may lead to diagnostic confusion with primary bladder cancer, particularly when presenting as a pseudotumoural lesion. In such cases, distinguishing between inflammatory and neoplastic processes can be challenging, often requiring a combination of imaging modalities and endoscopic evaluation. We report a misleading case of Crohn's disease revealed by pseudotumoural thickening of the bladder wall.

## Case presentation

A 63-year-old man, a former chronic smoker who had quit a year earlier, presented with lower urinary tract symptoms, including pollakiuria, urgency, and gross haematuria, evolving over several weeks. He had no known digestive disorders.

The results of the biological assessment showed an inflammatory syndrome, hypochromic microcytic anaemia, and a sterile urine culture (Table 1). 

**Table 1:** Laboratory findings at admission

Laboratory parameter	Result	Unit	Normal values
Hemoglobin	9.0	g/dL	12.0–16.0
C-reactive protein (CRP)	100	mg/L	<5
White blood cell count	6,590	/mm³	4,000–10,000
Urine culture	Sterile	-	

Renal and bladder ultrasound revealed an exophytic tissue lesion arising from the posterior wall of the bladder, measuring 61 × 39 mm with a longitudinal extension of 54 mm. The kidneys were normal, and the prostate volume was estimated at 60 cc.

**Figure 1:**
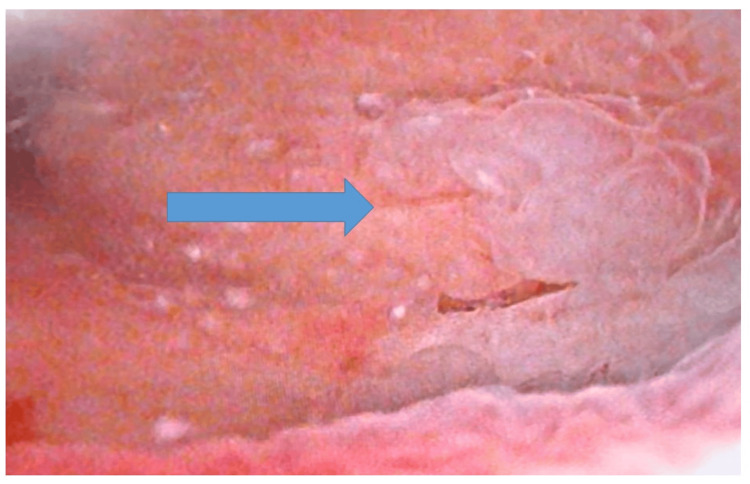
Cystoscopic view of a pseudotumoral bladder lesion without papillary features.

Cystoscopy followed by transurethral resection showed an inflammatory pseudotumoural lesion with no signs of malignancy (Figure 1). 

**Figure 2:**
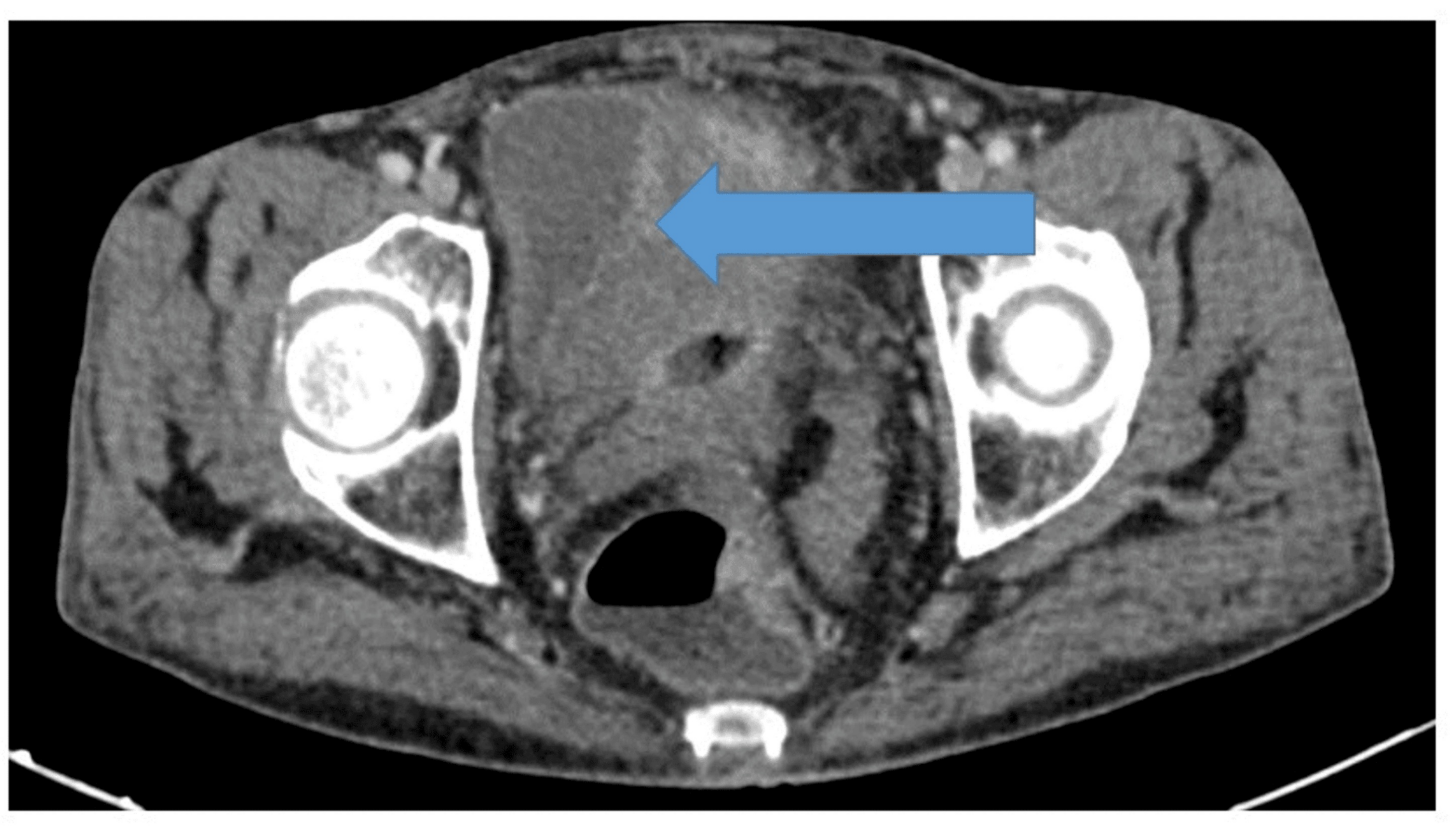
Axial CT scan showing a supravesical inflammatory collection in contact with the bladder dome, with associated bladder wall thickening.

Contrast-enhanced computed tomography showed inflammatory thickening of the terminal ileum and rectosigmoid colon with stenosis and multiple digestive fistulas. An inflammatory collection measuring 58 × 23 mm was identified above the bladder, in direct contact with the bladder dome, which showed marked reactive thickening of the wall with exaggerated enhancement of the mucosa, reaching a maximum thickness of 26 mm. No extravasation of air or contrast medium into the bladder was observed, ruling out an enterovesical fistula (Figure 2). 

Given the discordance between the initial suspicion of bladder malignancy and the inflammatory findings on histology and imaging, further multidisciplinary reassessment and gastroenterological evaluation were performed, leading to a diagnosis of Crohn's disease.

## Discussion

Urological involvement in Crohn's disease is rare and is most often associated with enterovesical fistulas [1]. However, isolated reactive thickening of the bladder wall without actual fistulous communication is rarely reported and represents a significant diagnostic challenge.

In this case, the bladder involvement resulted from its proximity to an adjacent suprapubic inflammatory collection secondary to fistulous Crohn's disease of the ileum and rectosigmoid colon. The absence of pneumaturia, faecaluria, recurrent urinary tract infections, and contrast passage into the bladder strongly ruled out an enterovesical fistula.

The patient's history of smoking and the presence of macroscopic haematuria strongly suggested bladder cancer, making the initial diagnostic suspicion of malignancy highly likely. Cystoscopy and transurethral resection were therefore necessary to exclude malignancy, ultimately revealing a pseudotumoural inflammatory lesion, as described in rare similar reports [2,3].

From a radiological perspective, delayed contrast-enhanced abdominal and pelvic CT plays a key role in differentiating between primary bladder tumours and secondary inflammatory lesions. In our case, the identification of adjacent digestive inflammation, suprapubic collections, and the absence of contrast agent passage into the bladder were key elements suggesting reactive bladder involvement rather than a fistula [4,5].

Furthermore, similar cases reported in the literature describe inflammatory pseudotumoural bladder involvement in Crohn’s disease as a rare entity that may mimic malignancy both clinically and endoscopically, often leading to diagnostic uncertainty and invasive procedures before definitive diagnosis [2,3].

It is crucial to recognise this atypical presentation quickly in order to avoid unnecessary aggressive urological interventions and ensure appropriate multidisciplinary management.

## Conclusions

Crohn's disease should be considered in the differential diagnosis of inflammatory masses of the bladder, even in the absence of an enterovesical fistula. Reactive thickening of the bladder wall secondary to adjacent digestive inflammation can closely resemble bladder cancer, particularly in patients with haematuria and a history of smoking. Although based on a single case, this observation highlights the importance of considering inflammatory conditions in atypical presentations to avoid misdiagnosis and unnecessary interventions.
